# Gleanings from Our Exchanges

**Published:** 1872-03-15

**Authors:** 


### Gleanings from Our Exchanges.

Dr. Thomas Addis Emmet, Surgeon to New York State Woman's Hospital, contributes an article on "Chronic Cystitis in the Female," to the *American Practitioner* for February, 1872. He dwells on the terrible results of cystitis if long continued. The surgeon is to discover, if possible, the cause of the cystitis ; it may be a rectocele, cystocele, prolaps or version of the uterus, hemorrhoids or fissure of the anus, in fact almost any disease affecting the uterus, vagina, rectum or digestive organs.

The first method of treatment that he recommends after these preliminaries have been attended to, is the frequent injection of large quantities of warm water into the bladder, through a double catheter. If the injection has been followed by much pain, it should be followed by a solution of morphine also injected into the bladder. If this treatment fails after a fair trial, cystotomy should be at once resorted to. The writer claims that the operation, if performed before the kidneys are involved, is as safe as any one in minor surgery. He has done the operation a number of times, with success in every instance. His method of operating is to introduce a sound into the bladder, where it is held by an assistant, with its point pressed firmly against the base, directly behind the neck. While the sound is held in this position, the projecting tissue is seized with a tenaculum, an opening is made, and the sound pushed into the vagina. Still holding the sound in the opening, one blade of the scissors is introduced into the bladder, and the opening is enlarged along the median line towards its base. The bladder is then to be washed, and the opening to be kept patulous at first, by the careful introduction of the finger ; but if that will not suffice, a hollow stud, made of glass tubing, half an inch in diameter, may be used. The flange which is within the bladder need only be narrow, not much more than a slight flaw ; the external flange should be wider ; the length of the stud should be only slightly greater than the thickness of the vesico-vaginal septum.

A New Pessary.—Dr. James Blake, of San Francisco, describes a new style of pessary in the *Pacific Medical and Surgical Journal* for February, 1872. The form of pessary is that of a Hodge or Hewett, the ends being cut out of sheet brass, and the sides of stout No. 1 watch spring soldered to the ends. After the parts are soldered, the instrument is to be varnished with India-rubber varnish, to prevent the corrosion of the steel by the sulphur in the rubber, which is next to be applied. Strips of sheet India-rubber slightly wider than the watch spring, are next to be fastened to their sides with varnish. Over this strips of thin sheet rubber, about a quarter of an inch in width, are to be wound so as to overlap slightly. About three layers are required. The first is to be wound tightly and the last loose. The ends are to be well stuck down, and the whole covered with about five coats of India-rubber varnish. The varnish is made by dissolving India-rubber in chloroform, and adding one-tenth of its bulk of a solution of asphaltum in chloroform, which has been allowed to settle before using.

Traumatic Tetanus Treated with Electricity.—Hamilton Griffin, M.D., reports in *American Practitioner*, Feb., 1872, a case of traumatic tetanus treated with electricity, in which recovery followed.

Tartrate of Iron and Potassa in Typhoid Fever.—Chas. H. Gordon, M.D., in *Pacific Med. and Surg. Jour.* for February, states that he has used tartrate of iron and potassa in typhoid fever with marked benefit. Dose—five grains twice a day, increased in third week to double the quantity.

Dr. Neil, of Philadelphia, in *American Practitioner*, Feb., 1872, reports a case of injury to the arm, in which gangrene and a very low condition appeared to follow the use of hydrate of chloral; on stopping the chloral and giving morphine, the patient improved rapidly.

Dr. D. T. Gillman advocates the administration of acids in certain cases of rheumatism, on the ground that the production of lactic acid is favored by an alkaline condition of the blood.—*Cincinnati Clinic, Feb.* 17, '72.

Dr. Ferrell reports excellent success in the treatment of acute dysentery by the use of ipecacuanha as an enema. His prescription is a drachm to six ounces of mucilage, repeated three or four times a day.—*Ibid.*

Dr. J. Bradley relates a method of treating certain inflammations by exhausting the air from around the lower extremities, thereby producing congestion of that part and abstraction of blood from the inflamed part, giving all the benefits of blood letting, and at the same time saving the blood. He has tried it in pneumonia, with excellent results. —*Ibid.*

Billroth on Ovariotomy.—This eminent surgeon, in his "Reminiscences," published in the *Wiener Med. Wochenschrift*, says of ovariotomy:

First of all, surgeons must dismiss from their minds that ovariotomy is a dangerous operation; and, through the medium of well-informed practitioners, this conviction must make its way with the public. After ovariotomy, skilfully performed according to the rules of art, recovery is the general rule, and a fatal issue the constantly diminishing exception. Comparing it with some other operations, ovariotomy, taking the mass of cases, is shown by statistics to be less dangerous than amputation of the thigh, disarticulation of the shoulder and hip-joints, or excision of the hip or knee. Its danger is about the same as that of amputation of the arm, excision of the shoulder, partial excision of the jaw, lithotomy in the young, and similar operations. We must, however, perform ovariotomy strictly according to the rules laid down by the English operators in their classical works; and only after having attained the same results should we venture to practically put into force our own ideas, in order to improve upon these. I had the good fortune to see Spencer Wells operate upon two complicated cases, and from them, as well as from oral communication with this remarkable man, I learned much. I constantly follow his precepts, knowing that he has long since thoroughly thought out and tested all that can happen to myself. I shall willingly regard myself during my lifetime as his scholar; and contented shall I be if it falls to my lot, by means of this operation, to snatch from certain death one-half of the number of lives he has been enabled to save.

Up to the present time I am tolerably contented with my results. I give here a short account of them, in order to encourage the performance of these operations, and especially to inform the colleagues into whose hands these lines may fall that I have, personally, no reason for supposing that the results attendant upon ovariotomy will be less cheering in Vienna than they are in London. Hitherto I have performed it nine times, and of these patients only two have died, giving, therefore, only a mortality of 22.2 per cent. The first four cases recovered one after another; then two fatal cases occurred, to be followed again by three recoveries. The first case is related in my Zurich "Chirurgische Klinik," and the second, third and fourth cases in the "Chirurgische Klinik," published at Vienna in 1868.—*New York Medical Journal, Feb.*, 1872.

Chloral in cod-liver oil is said to render it much less nauseous, and prevents the night-sweats of the phthisical patient, induces sleep, and creates appetite. The pure chloral-hydrate crystals may be added to cod-liver oil in the proportion of 10 grains of the former to 190 of the latter.—*Ibid.*

Dr. C. C. Ritchie, in *London Practitioner*, states that the hypodermic injection of five grains of ergotin, will stop the bleeding in hæmoptysis speedily, when all other means have failed.—*Ibid.*

Compressed air is said to cure whooping-cough.

OPHTHALMOSCOPE IN BRIGHT'S DISEASE.—In a paper read before the Boston Society for Medical Observation, Dr. Williams relates two cases of Bright's disease in which the autopsy proved conclusively the correctness of the diagnosis of Bright's disease which had been made by the ophthalmoscope. The disease of the kidneys was not suspected before the examination with the ophthalmoscope. He says that in cases of kidney disease, accompanied by changes in the retina, the prognosis was always unfavorable.—*Boston Medical and Surgical Journal, Feb.* 15, 1872.

ABSENCE OF VAGINA.—Dr. Curtis reports to Boston Obstetrical Society, a case of complete absence of vagina in a girl 16 years of age, there being distension of abdomen, with pain at each probable menstrual period. An operation was performed, resulting in the discharge of a thick chocolate-colored fluid. Pain and abdominal tenderness followed, and the patient died about two weeks after the operation.—*Ibid.*

PERIODICAL HEADACHES. — Dr. Bradnock reports a method of treating periodical headache, which he claims to be original as well as effectual in curing the disease. He enumerates several of the symptoms, and claims that these all point to either active or passive congestion of the brain or its membranes.

The treatment divides itself into two parts —first, what is proper to be done during the attack; second, what is proper in the interval. He claims that there is always constipation of the bowels, consequently, if he begins treatment during the interval, he gives one or two of the following pills :

℞ Mass hyd.
Ext. coloc. com.
Pulv. aloes soc. aa xi.
Pulv. ipecac, gr. vi.
M. Ft. pil., No. xij.

To be followed by one (1) drachm of sulphate of magnesia. Then he begins with three drops of liquor potassa arsenitis, to be taken in a drachm of water after each meal.

If the patient is delicate and complains of coldness of the extremities during the attacks, and frequent chilliness during the intervals, he substitutes the following:

℞ Liq. arsenicalis hydrochloric, ʒ ss.

Quiniæ disulphat, gr. xij.

Lig. ferri perchloride, ʒ ij.

Aquæ, ʒ vi.

M. (S. One tablespoonful in a wine-glassful of water, twice a day, after meals.)

Whichever one of these is given, it is to be interrupted once in three weeks, and the first prescription given.

When the attack begins he places the patient in a chair, with the head elevated, the feet in a hot mustard bath, the hands in warm water, and a bag of ice on the head, if it can be borne, and gives the following prescription:

℞ Potasii bromid, ʒ vi.

Ammon. bromid., ʒ ij.

Potasii iodide, gr. vi.

Infus. columbo, f ℥ iij.

M. S. One dessertspoonful in an ounce of water.

This treatment persevered in three or six months, he claims, will cure nearly every case. —*Buffalo Med. and Surg. Journ., Feb.*, 1872.

